# Living bridges using aerial roots of *ficus elastica* – an interdisciplinary perspective

**DOI:** 10.1038/s41598-019-48652-w

**Published:** 2019-08-22

**Authors:** Ferdinand Ludwig, Wilfrid Middleton, Friederike Gallenmüller, Patrick Rogers, Thomas Speck

**Affiliations:** 1 Professorship for Green Technologies in Landscape Architecture, Technical University of Munich TUM, 80333 Munich, Germany; 2 Plant Biomechanics Group, Botanic Garden, University of Freiburg, Freiburg im Breisgau, 79104 Germany; 3 Independent Researcher, 34, Minquil Drive, Newark, DE 19713 USA; 4 Freiburg Materials Research Center (FMF), Freiburg im Breisgau, 79104 Germany; 5 Freiburg Centre for Interactive Materials and Bioinspired Technologies [1], Freiburg im Breisgau, 79110 Germany

**Keywords:** Ecology, Plant sciences

## Abstract

Here we report on a pilot study of the Living Root Bridges (LRBs) in the Indian State Meghalaya, which are grown with aerial roots of *Ficus elastica*, a facultative hemiepiphyte developing abundant aerial roots. Locals use these aerial roots to build living bridges, which strengthen themselves over time due to adaptive secondary growth and their capacity to form a mechanically stable structure via inosculations. An extensive inventory of LRBs in Meghalaya including data of location, altitude, approximate age and bridge length was performed in field studies. Root morphology was characterised by measurements of cross-sectional area and shape-related parameters and analysed in relation to the orientation of the roots. LRBs are found to occur mainly in the mountainous limestone rainforests where *F. elastica* may be native or traditionally cultivated. They cover an altitude range of 57–1211 m a.m.s.l. and display a length of 2 to 52.7 m. Some bridges are several hundreds of years old. Horizontally and vertically trained roots differ significantly in shape and cross-sectional area when approximately even-aged roots are compared. The results are discussed from an interdisciplinary perspective, considering the adaptive traits in the natural life cycle of *F. elastica* and possible applications in living architecture (Baubotanik).

## Introduction

**Figure 1:**
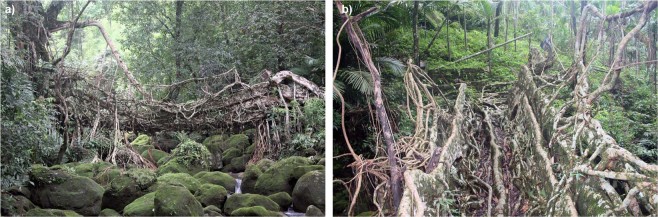
(**a**) Ummonoi Bridge, with a span of 7 meters, is a typical example of a mature living root bridge. Other bridges have spans up to 53 meters. (**b**) is taken from the centre of the bridge, looking towards the right-hand end of (**a**). (bridge #57 in Table S1 in Supplementary Information).

The special features of growth and mechanical properties of the aerial roots of *Ficus elastica* have been well known and utilised for centuries by the indigenous Khasi and Jaintia people in the subtropical moist broadleaf forest ecoregion of Meghalaya. The region is dominated by steep valleys leading from the Shillong Plateau to the Bangladesh floodplain. Here the inhabitants of isolated villages devised a way to construct bridges with living aerial roots of *F. elastica* in order to cross monsoon-swelled rivers^1–3^. The bridge-building technique obviously takes advantage of the mechanical strength of living aerial roots of *F. elastica* and their natural tendency to anastomose and form a mechanically stable structure via inosculations (Fig. 1).

The technique of using aerial roots of *F. elastica* to form bridges is a unique example of botanical architecture grown without the tools of modern engineering design. While there is quite a number of examples of living architecture worldwide, LRBs provide the only known example of repeated, predictable use of tree growth for structural purposes (see Ludwig’s, 2012 historical introduction^4^). Until recently it was thought that only a handful of such bridges existed^1^, and investigations into their structure, distribution and morphology are accordingly limited. Chaudhuri *et al*. and Shankar describe the societal setting of the bridges and their construction methods^1–3^. Many media platforms have covered the LRBs and the communities that build them, ranging from blogs^5^ to TV documentaries^6,7^ and books^8,9^. With regards to *Ficus* species in general, Shanahan offers a summary of their worldwide cultural significance^10^.

Commonly, a *F. elastica* cutting is planted on one bank of a canyon or river. After reaching an adult stage, aerial roots (referred to herein also as ‘roots’, unless specified as ‘subterranean’) emerge from the branches and are wound onto and directed across a deadwood framework (mainly bamboo). This anthropogenic process takes roots that generally hang vertically down and uses them horizontally to cross the river. When they reach the opposite bank, the roots are implanted. They then shorten, start to thicken and produce daughter roots, which are trained (wound and directed) similarly. Generations of builders contribute over decades or centuries to bridge structures, rarely with a clear or consistent plan. Through the close intertwining of roots, inosculations can be initiated to form a densely interwoven framework-like structure. Alternatively, the initial root(s) used in the bridge can be allowed to grow unaffected and dominate the structure. The addition of handrails, a second deck, underpinning struts, or other features can further influence the bridge’s structural system. This combination of continuous growth and maintenance leads to a high level of complexity in each bridge, making simple mechanical analysis unfeasible. LRBs show a very wide variety of structural typologies, with various aspects of particular bridges resembling characteristics of suspension bridges, cable-stayed bridges, arches, trusses, and simply-supported beams.

*Ficus elastica* Roxb. ex Hornem. (Moraceae) is a facultative hemiepiphyte and belongs to the group of “strangler figs”. In these species germination of the bird-dispersed seeds in the canopy of a host tree is followed by an epiphytic growth phase. In later ontogenetic stages aerial roots are developed, which grow downwards through the air from branches of the host tree or along the host tree’s stem and finally root in the ground. In older hemiepiphytic *Ficus elastic*a individuals the aerial roots anastomose, form inosculations (natural grafts) and build a scaffold around the host tree’s stem. Canopy shading, root competition and the prevention of transport in the outer vascular tissue of the host tree’s stem by the network of the strangler fig’s aerial roots may ultimately cause the death of the host tree. After decomposition of the former host tree a hollow cylinder composed of the network of partly fused aerial roots supports the surviving strangler fig.^11,12^. Based on a study of Australian strangler figs Richard and Halkin^12^ postulate that the presence of a strangler fig can also have beneficial effects on a host tree by shielding it from cyclones.

As known from other strangler plant species, also *Ficus elastica* can start growing from the ground under certain conditions, e.g. from cuttings or germinating on boulders, cliffs and rocks which are common to the Khasi and Jaintia Hill regions. These specimens, which grow without the support of a host tree, develop abundant aerial roots, similar to their hemi-epiphytic counterparts. In general, the ecology of *F. elastica* is not well understood. Nonetheless, more detailed information is available on effective seed dispersal^13^, germination^14^, physiology^15^ and adult ecology^16^ of fig species.

An increasing amount of information is available on the mechanical properties of roots and their mode of anchorage in the ground for a variety of herbaceous species^17–24^ and woody species^25–31^. However, only little is known on aerial roots in woody hemiepiphytic species. Eskov, Hinchee and Tenorio *et al*. report on growth properties and anatomy of aerial roots in herbaceous hemiepiphytic species within the Araceae^32–34^, and Patino *et al*. have analysed growth rates and mortality rates of aerial roots in different tropical Araceae and Clusiaceae^35^.

Scientific information on development, functional morphology and mechanical properties of aerial roots in strangler figs are scarce. Zimmermann *et al*. (1968) have studied the growth of aerial roots in *F. benjamina* and shown that after anchoring in a substrate the aerial roots temporarily produce tension wood, which causes them to contract^36^. The production of tension wood in the whole circumference of the roots shortens and strains them in the first developmental phases and helps in pressing neighbouring roots with relatively high forces on each other (see also Abasolo *et al*.^37^). Therefore, tension wood formation is vital to the inosculation process used in constructing living root bridges (hereinafter referred to as, LRB”).

Inosculations between roots, branches or stems are reported to develop naturally in several species. Millner (1932) describes the process in detail for *Hedera helix*. In this species at first the phelloderm merges, followed by the xylem, subsequently forming a continuous cambium layer and then a common growth ring^38^. A similar process has been described by Ludwig for several European trees species with thin bark^4^. To the authors‘ knowledge, no information is available on the formation process and the anatomy of inosculations in aerial roots of *F. elastica* (developed either under natural conditions on a host tree or in a living root bridge).

For the present study three survey expeditions to the Khasi and Jaintia Hills of the Indian state of Meghalaya have been carried out in 2015, 2016 and 2017, in order to (1) create an extensive inventory of the so far only fragmentarily documented living bridges in the region, (2) geolocate these bridges, (3) characterise their structural properties, (4) gather information on their history and maintenance, and (5) analyse the morphology of a selection of structurally important aerial roots of *F. elastica* in these LRBs. This approach is designed as a pilot study and aims to provide a basis for further analyses of the functional morphology and biomechanics of *F. elastica* roots and their ecological significance, and for further understanding structure, mechanics and development of LRBs. The choice of studying such aerial roots grown under artificial conditions in a living bridge offers the essential advantages of (1) being able to investigate roots of at least partly known age (which due to the lack of growth rings in the secondary xylem is not possible in field work on living specimens grown under natural conditions) and (2) simultaneously allows for studying roots after several decades of growth under mechanical loading (which to this extent cannot be done in greenhouse experiments under controlled conditions).

For analysing the morphological and possible structural adaptations of the roots, we separated them into two categories. Since loading regimes cannot be determined in detail within the complex LRB structures, for this pilot study we categorize roots by orientation and compare horizontally- and vertically-oriented root sections. This is based on the simplifying assumption that horizontally oriented roots (as beamlike elements) are more likely to undergo bending stresses while horizontal ones are more likely to experience axial compression or tension stresses.

In a broader context the study also aims at gathering information for a future implementation of the functional principles of growth in aerial roots of *F. elastica* in concepts of botanical architecture (Baubotanik, see e.g. Ludwig^4^ and Ludwig *et al*.^39^). Due to their possible capacity to react to mechanical loading with adaptive secondary growth, the development of inosculations and the formation of -tension wood, enabling them to fulfil a task as a tree-supporting structural element despite their evolutionary design as roots, the aerial roots of *F. elastica* (and of strangler figs in general) can be considered as a unique concept generator in the field of botanical architecture.

Here we present the first extensive inventory of living bridges built with aerial roots of *F. elastica* in Meghalaya as well as their geographic distribution and type of maintenance. We furthermore analyse and discuss data of aerial root morphology in the studied LRBs in relation to their size and, where possible, to their age. Thus, the study aims to deepen the knowledge of LRBs, linking it with relevant fields such as plant biomechanics, morphology and engineering.

## Methods

During the surveys, bridges were located through work with guides across the Meghalaya region who established contact with different local communities involved in building and maintenance of LRBs. Photographs, measurements, geolocations, and interviews were taken with these guides, who also acted as translators. The interviews were transcribed in note form. Since the interviewees live in very small places with closely connected families, and because each LRB and its individual history is highly specific, it was not possible to represented the interviews in a fully anonymous way. To protect the identity of the interviewees, the interviews therefore are not reported here.

Geolocation and altitude data were determined for 72 of the 76 bridges using a combination of GPS, A-GPS, and GLONASS (with an expected accuracy of 15–30 meters) during each of the three surveys from 2015 to 2017. These were compared against a range of online mapping services^40–42^ to provide more accurate locations, suitable for reference to current geological, terrain, and forest cover data. A similar procedure was used to locate rural village centres. QGIS v2.18 was used to display the bridge coordinates with reference to WGS 84. Topographic data, precise to 90 m, was taken from the SRTM database^43^. Tree cover data was taken from Hansen *et al*.^44^, coordinates of major towns from OpenStreetMap^40^.

Bridge lengths were determined using a measuring tape and laser distance measure. The length of a LRB is defined here as the distance over which a person crossing the bridge will be suspended above the ground solely by the structure itself. This distance was ascertained by first locating the point at which the structure leaves the ground on one side of a river bank, then finding the place at which it reconnects on the opposite side, and determining the distance between these two points. The exact points are not always entirely clear, and might differ slightly from the right and left sides of the bridge. There are often components which are structurally integral to a given root bridge but are not part of the actual span of the bridge. These have not been measured. In the instances of root bridges with two independent spans, each span has been measured separately. 73 lengths of 70 bridges have been measured.

Many characteristics of the bridges are not standardised throughout the region, and are as yet not widely described. In order to gain a deeper qualitative understanding of these areas, interviews were conducted. Specific questions related to the construction and growth processes, use, and ownership yielded helpful information. Bridge age data was also collected through interviews. Confident age estimates were given for 15 bridges (see Table S1).

As site conditions may influence the growth considerably we present age data of roots collected only within one particular bridge in this. Root age information is based on the oral account of one interviewee who, having been the sole builder of this bridge, identifies clearly the different stages of his work. In total the age of 57 roots integrated in this bridge was determined. Aerial root inosculations were documented with photographs and the techniques used in their formation were discussed with local guides and bridge-builders.

When investigating structurally important roots, it was found that each bridge, as well as each constituent root, is very complex. Fusing, branching, and changes of shape, size and direction, lead any systematic survey of root geometries to lack detail. As a result, a number of roots were selected to give a fair representation of the geometries of roots that appeared structurally important to each bridge while being measurable in the field.

Geometrically measurable, structurally important roots were determined through three criteria, gauged visually and with a tape measure. The root had to appear to (1) support dead or live load, supporting the tree, other roots, or pedestrians; (2) be straight along its length: height change of the root’s perceived centroid (visual observation) no more than one tenth of the root’s length; and (3) have a length of consistent cross-sectional dimensions at least five times greater than the largest cross-sectional dimension. Roots from a variety of places in bridges (deck, rails, supports) were measured. Five such roots can be seen in the Ummonoi bridge (Fig. 1b).

Root dimensions were taken by digital Vernier callipers (below 150 mm) and tape measure (above 150 mm). Root lengths up to 7.5 m were measured by tape measure and laser distance measure above 7.5 m. Cross-sectional dimensions were taken halfway along the root’s length, at the mid-point between where the root originates (from the tree trunk or another root) and where it branches or inosculates and the cross-sectional dimensions are changed.

**Figure 2:**
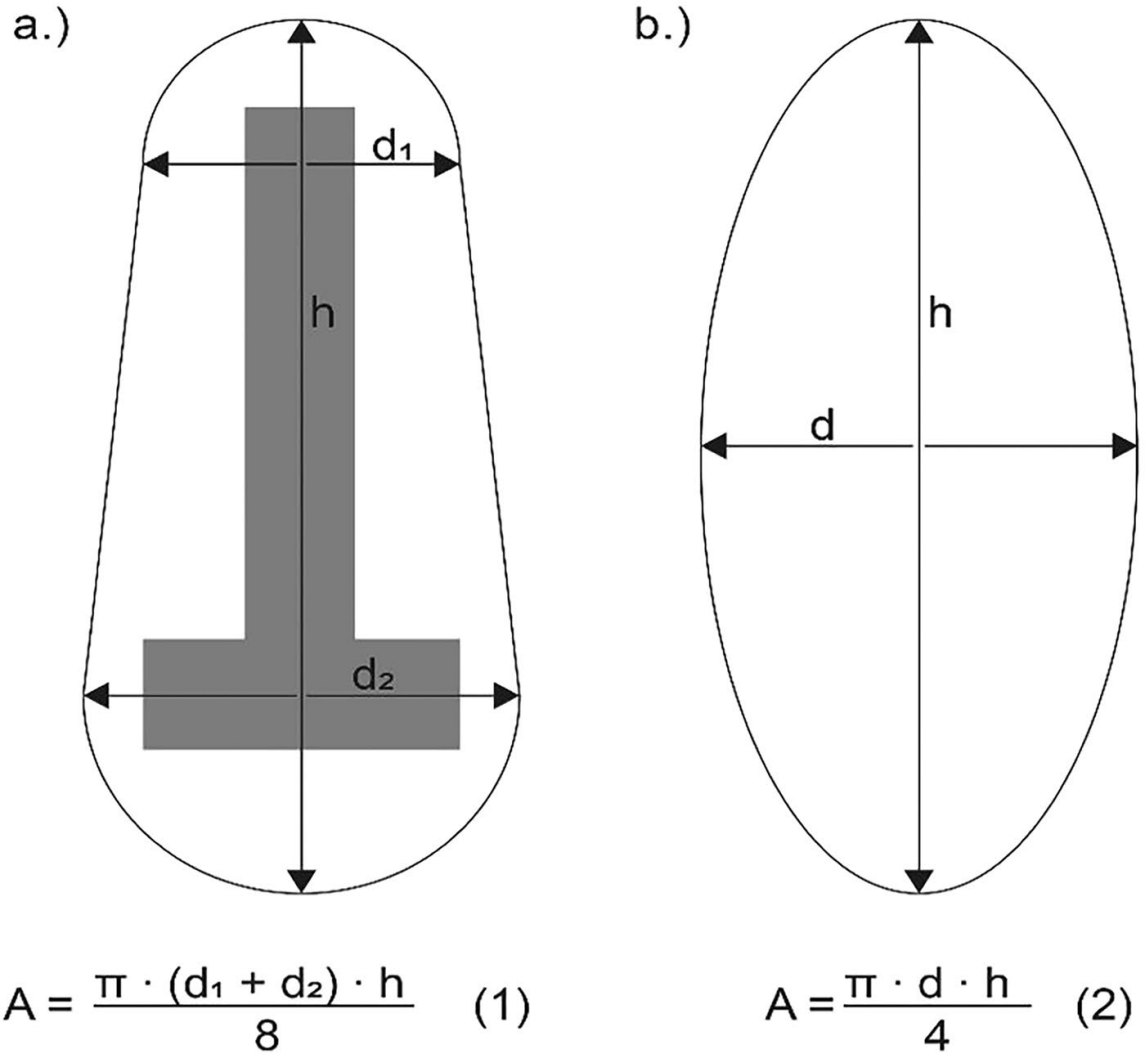
Parameters measured in roots with a T-shaped cross-section (**a**) and with an elliptical cross-section (**b**) and equations of the cross-sectional area *A*. In grey the idealized inverted T-shape. Equation (1) slightly underestimates the CSA but was used here for reasons of compatibility with elliptical cross sections.

For roots with an approximately T-shaped cross-section (inverted T) the ‘T-ratio’ was calculated as the ratio between the largest and smallest widths in the minor axis (d1/d2, Fig. 2a), following Nicoll & Ray^45^. Cross-sectional area *A* was approximated by Equation (1) (Fig. 2a). For roots with an – idealized – elliptical cross-section the ratio of height h to width d was calculated as a measure for the deviation of a circular shape (h/d, Fig. 2b). Per definition, h/d equals 1 in circular cross-sections. The cross-sectional area of roots with an elliptical cross-section was calculated according to Equation (2) (Fig. 2b).

Statistical analyses of root morphology in roots of different age were carried out using Python 3.4.5 (Pyzo 4.3.1 binary). All data sets were tested for normality by D’Agostino-Pearson test (α = 0.05). As not all data sets are distributed normally, median and interquartile ranges were calculated and plotted in box plots. Data were further tested by Mann-Withney U test (α = 0.05). The statistical significance is indicated by using the p-value.

As described in the introduction, we focus on horizontally and vertically oriented roots respectively root segments. Though this is a simplification (as can be seen in Fig. 1b) many roots fit well into these two categories. All roots used in the analysis are within ±30° of the horizontal or vertical. Where a root is growing at a deviation of more than 30° from the horizontal or vertical, data has been omitted from the present analysis. This excludes diagonals – which undoubtedly may play an important structural role in frameworks – but allows for a first analysis of roots as structural elements in these complex systems. A total of 149 roots or root segments were measured.

## Results

**Figure 3:**
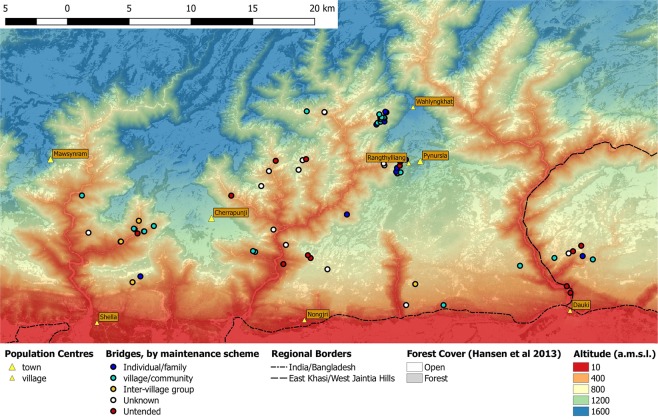
Map of the Khasi and Jaintia Hills illustrating bridge locations maintenance types, altitude (based on SRTM data according to Jarvis *et al*.^33^ and forest cover (as mapped by Hansen *et al*.^34^).

**Figure 4:**
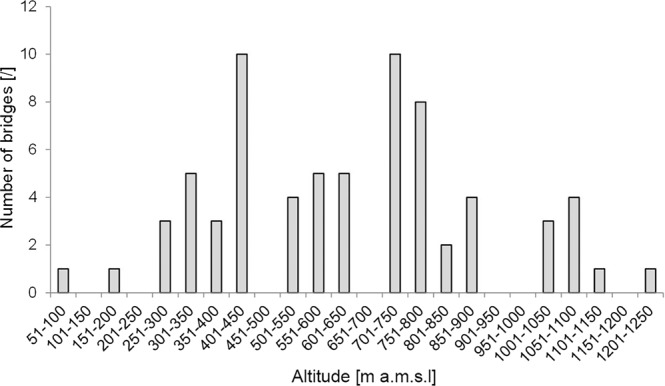
Histogram of bridge altitudes at 50 m intervals, using A-GPS and GLONASS data collected for 71 bridges.

Figure 3 shows the distribution of LRBs as documented between 2015 and 2017. 68 of the 71 geo-located bridges are located in the rainforest valleys. Two bridges (#14 and #58 Table S1 Supplementary Information) are on the plateau but less than 400 m away from a valley, one is on the plateau at a distance of 1 km from a valley (#5 Table S1 Supplementary Information). A closer look shows a pattern of clusters of bridges formed around villages as well as isolated bridges dispersed through the region. The map reveals that most of the clustered ones are well maintained while many of the dispersed ones are untended. Figure 4 shows the distribution of altitudes at which the bridges are grown. The majority grow between 250 and 900 m a.m.s.l., while there are clear outliers between 0 and 100 m and 1000 and 1250 m.

Through the conduction of interviews, information on bridge use, maintenance and history could be recorded. All bridges documented here were grown as part of a river crossing path between villages or from a village to cropland or to markets. Maintenance consists of a variety of techniques: removal of mosses and epiphytes, pruning and tying of roots, laying of material (stones, soil) on the path, clearing of the associated path. Through interviews and observation, it was found that maintenance is done by individuals or families (12 of 75 bridges), shared amongst a village community (25 bridges), or by a consortium of several communities (8 bridges). Maintenance is conducted on another 14 bridges, though the maintainer is unknown, and not conducted on 16 bridges (untended) (Fig. 3).

**Figure 5:**
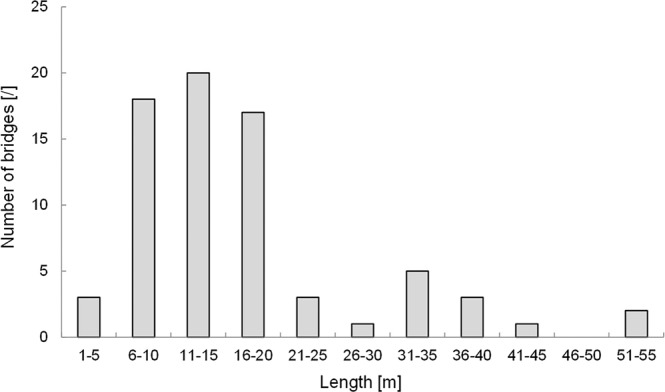
Length of 70 LRBs (with 73 measured lengths).

The length of the bridges varies between 2 and 52.7 m. 58 of 73 measured lengths, or almost 80%, are shorter than 20 m, with frequencies above a length of 20 m falling off sharply (Fig. 5). In some cases, the aerial roots reach more than 30 metres away from the parent tree.

**Figure 6:**
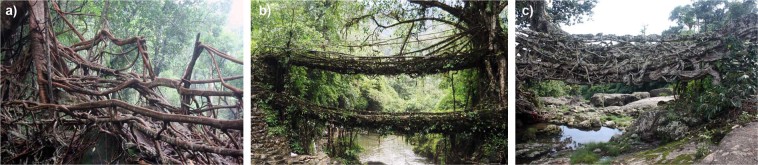
(**a**) Partial view of Siej bridge (#50). The bridge is an example of bridges that are grown by currently living people. (**b**) According to the statement of residents/users involved in the maintenance the bridge in Nongriat village (#6) is estimated to be ca. 200 years old, an example of bridges started by known ancestors. (**c**) The bridge linking the two sides of Nongbareh village (#62) is part of a vital route. It is estimated to be as old as the village, probably hundreds of years old.

Figure 6 shows three bridges of different age estimates which represent examples for three categories. Bridges grown by currently living people in the region; bridges built by known ancestors, generally no more than five generations old; and bridges known to be very old but with no known histories other than those tied to village histories (see age estimates in Supplementary Information Table S1). The interviews suggest that many bridges grown in the past were destroyed since, probably by floods, fires, and landslides.

**Figure 7:**
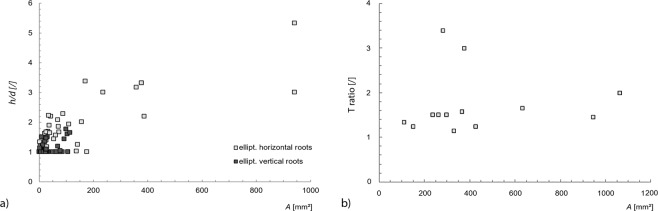
Root geometry. (**a**) Ratio of height to width (h/d) and cross-sectional area (A) of horizontally and vertically trained roots with an elliptical cross-section. (**b**) T-ratio and cross sectional area (A) of horizontally trained roots with a T-shaped cross-section (vertically oriented roots did not show T-shape).

In roots with an approximately elliptical cross-section the ratio of height to width (*h/d*) increases in both horizontally and vertically oriented roots with increasing cross-sectional area *A* (Fig. 7a). However, within the range of comparable size (cross-sectional area <140 mm²) the elliptical ratio is significantly smaller in vertical roots than in horizontal roots. 72% of the measured vertical roots have an approximately circular cross-section with an elliptical ratio = 1 (Fig. 7a).

In horizontally orientated roots the T-ratio does not vary significantly with increasing cross-sectional area (Fig. 7b). Whereas the majority of the measured roots with a T-shaped cross-section have a T-ratio of 1.2 to 1.7, two roots had a considerably more pronounced T-shape with a T-ratio of 3.0 and 3.4 (Fig. 7b). Among vertically oriented roots no T-shaped cross-sections were observed.

**Figure 8:**
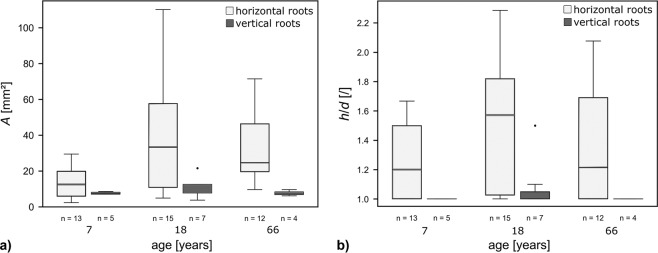
Geometry of horizontally and vertically trained roots developed approximately 7, 18 and 66 years after their implementation in the bridge #50 (see Tables S1 and S2 in Supplementary Information). (**a**) Ratio of height to width (*h*/*d*), (**b**) mean cross sectional area (*A*). n = sample size.

For 57 roots of one bridge the approximate year of their implementation in the bridge could be determined. They fall into three age groups (ca. 7, 18 and 66 years old). In each age group the gain in cross-sectional area (*A*) and the ratio of height to width (*h/d*) are higher in horizontal than in vertical roots (*A*: 7 year old roots: p = 0.495, 18 year old roots: p = 0.052, 66 year old roots: p = 0.004, Fig. 8a, *h*/*d*: 7 year old roots: p = 0.031, 18 year old roots: p = 0.026, 66 year old roots: p = 0.061, Fig. 8b). No significant difference of *A* and *h*/*d* is observed between roots implemented 18 years ago and roots implemented 66 years ago, although the values vary over a wide range in each group (Fig. 8).

**Figure 9:**
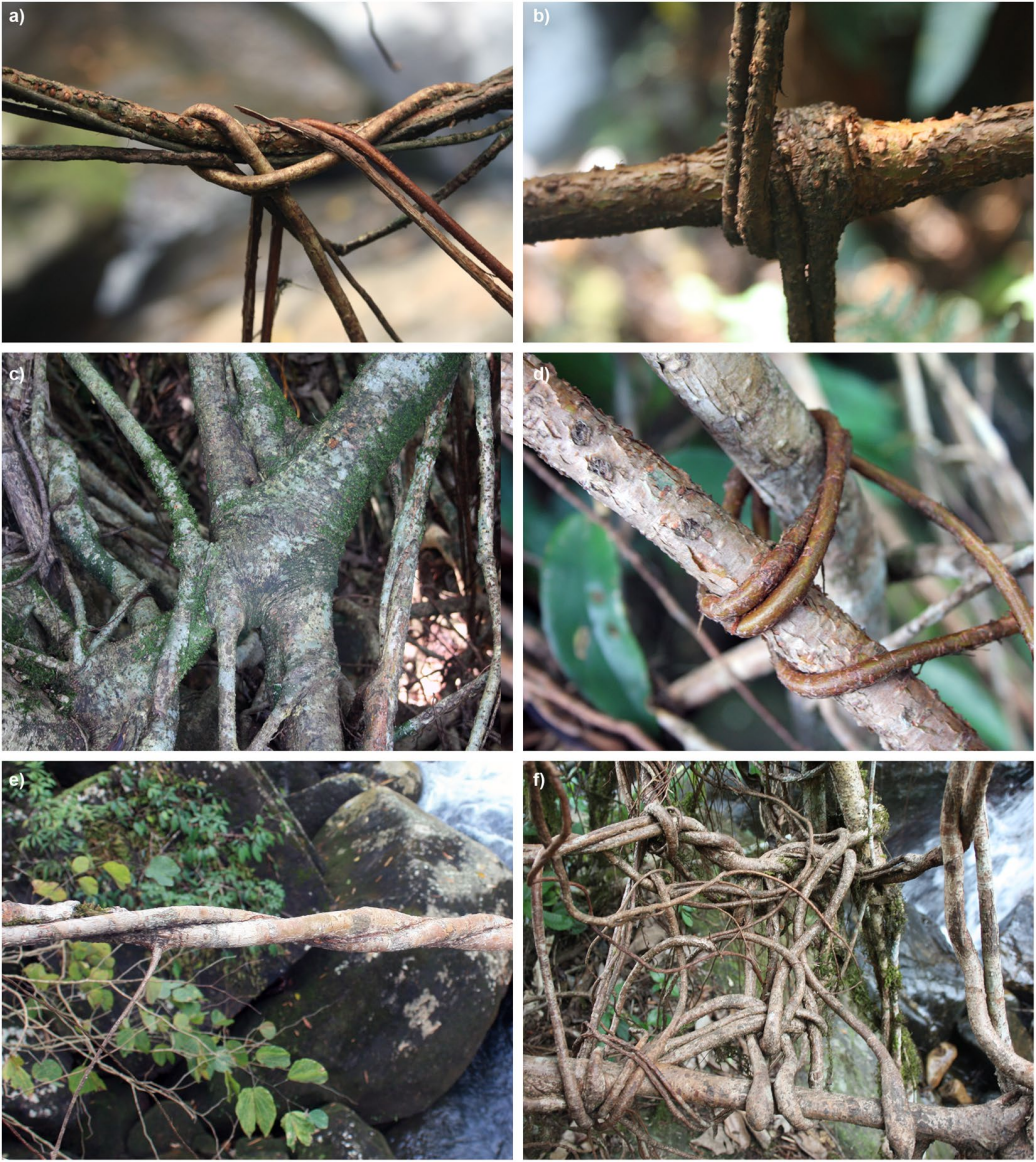
(**a**) Man-made inosculations in LRBs. (**a**) young nodal connection, (**b**) a similar connection after tightening and the beginning of inosculation, (**c**) a fully established inosculated node, (**d**) a young root used to tie together two older ones, (**e**) a typical example of a twisted linear connection with first signs of inosculation, (**f**) a structure with thickened roots showing no inosculation.

A wide range of inosculations were documented from 48 bridges. According to their geometry, nodal connections and linear connection can be distinguished. The simplest nodal connections are formed when two roots just touch each other crosswise and inosculate. In other cases two or more roots are knotted or twisted together at a specific point, forming a more or less precise node (Fig. 9a). Linear connections are generally formed by roots twined together over a long section, forming a cord (Fig. 9e). The structure of a bridge is built upon a combination of these two connection types. In a first step free hanging roots are connected to a quite loose, network like structure (Fig. 9a). After the roots have anchored to the ground, they visibly come under tension, straighten and thicken. Induced by this tension and thickening the roots are pressed together at the connection points and start to inosculate (Fig. 9b, see Zimmermann’s discussion of tension wood in aerial roots^36^. In a third step the roots form a unified whole at the connection points with a smooth and homogeneous surface that makes it often impossible to identify the original root sections (Fig. 9c). In addition to these joints, which are created by the connection of young roots of similar ages, young, free-hanging roots are used to tie together older ones, often leading to inosculations both between the old roots and between the young and old roots (Fig. 9d). In some cases thickening roots did not show any signs of inosculation (Fig. 9f). In other cases, trained roots died before they reached the ground.

## Discussion and Conclusion

In the Khasi and Jaintia Hills of Meghalaya, inhabitants of isolated villages have devised a practical and sustainable way of constructing living bridges grown with aerial roots of *F. elastica* in order to cross monsoon-swelled rivers. Where bridges made from bamboo or wood would rot and be easily swept away, and structures made from steel or concrete would be expensive, requiring outside investment and maintenance while also rusting quickly and being easily damaged, the villagers made use of the mechanical strength of the aerial roots of *F. elastica* and their natural tendency to anastomose and form a mechanically stable network via inosculations, able to withstand mechanical stresses. The tradition of building LRBs is thus based on the presence of *F. elastica*. The species was first documented in Meghalaya in the early 19^th^ century^46^ but its detailed distribution within the region remains unclear. In particular, no previous studies directly address *F. elastica* populations in the Khasi or Jaintia Hills. Research in the surrounding forest areas of Northern Meghalaya^47^, Mizoram^48^, and West Bengal^49^ find *F. elastica* present but not dominant and relatively sparsely dispersed. Corner^50^ suggests that *F. elastica* is native to the limestone areas in North-East India (including Meghalaya) and Myanmar, with outlying relics in Malaysia, Java and Sumatra^50^. In contrast, Harrison *et al*. propose that the species is native to western Java, southern Sumatra, Peninsular Malaysia, western Thailand and North Burma but not to North-East India^51^. Furthermore, it is documented that *F. elastica* has spread into new areas by cultivation for rubber production, both before and during colonial times^52–54^. The inventory of LRBs in Meghalaya presented here gives evidence for a further local anthropogenic influence on the distribution of *Ficus elastica* exceeding the use as latex source. LRBs are found to occur most commonly in the mountainous limestone rainforests to which *F. elastica* is either native^50^ or at least traditionally cultivated for rubber production^52–54^. While it is possible that trees used in LRBs were spread naturally, many mother trees of the bridges clearly show a solid trunk, suggesting that they did not grow epiphytically and probably were planted. The present study with its focus on LRBs cannot answer the question of whether *ficus elastica* propagates naturally in the area or not. Therefore further studies with a broader focus are needed, including molecular techniques, an inventory of fruit-bearing specimen and seedlings developing as epiphytes on other tree species (compare Chantarasuwan *et al*. 2016, Harrison *et al*. 2017)^51,55^.

Obviously, and as can be seen in Fig. 3, LRBs are mainly located in steep valleys where there is a need to cross canyons and rivers. Simultaneously stable village populations in and around valleys are needed for the construction and long-term maintenance of the bridges. So far, data do not allow for a statement on the time needed from beginning of construction to full functionality. This time span depends on a number of factors, not least, of course, the desired span of the bridge. The example of still not fully functional bridge number #50 with its growth time of already 66 years gives a first idea (Supplementary Information, Table S1). Environmental factors like sunlight, nutrients and water supply certainly have a great influence, too. Future investigation may shed light on how these factors interact and how they affect the development of a bridge.

The first step of the construction of a LRB being the planting of a *F. elastica* cutting at the location of the future bridge means that a *F. elastica* individual is introduced at each bridge location by human activity. In this context the LRBs can be considered as both a man-made technology and a specific kind of plant cultivation which is implemented across canyons and rivers between villages, markets, and croplands. Observations of spreading up to 50 m across a canyon by large *F. elastica* trees implemented in bridges that are probably hundreds of years old corroborate that the species can successfully colonize an area exceeding their point of insertion at a river-crossing path by vegetative propagation. The extent of this process and a potential establishment of generative reproduction have to be elucidated in further.

The aerial roots of *F. elastica* forming LRBs display significant differences in shape (and also of size when approximately even-aged roots are compared) according to their orientation within the bridge. Root thickening and development of noncircular cross-sections (elliptical and T-shaped) may be genetically predetermined and/or develop as modifications in reaction to stress or strain regimes^45,56^. As inverted T-shaped cross-sections were observed exclusively in horizontally trained aerial roots and elliptical cross-sections were found to be significantly more pronounced in horizontal roots than in vertical roots (of comparable age) the development of such noncircular cross-sections can be considered as a phenomenon of adaptive secondary growth. Orientation towards gravitational force and/or the type of mechanical loading may be among the abiotic factors controlling these growth processes. Horizontal roots of *F. elastica* with a T-shaped cross-section exclusively showed increased growth on the lower side, leading to an “inverted T-shape”. Smith (1972) presents a range of positive and negative selectors for elliptical growth of buttress roots in the tropics, including the more efficient use of wood by bending load as a positive and the higher surface area of the buttress as a negative selector^56^.

Horizontal beams subjected to gravitational bending moment experience tension stresses on their lower side and compressions und their upper side. This also holds for roots or root segments supported at two points, e.g. their point of origin and the point of first branching or the point of entering the ground. Nicoll and Ray suggest that morphological adaptations in subterranean roots are linked to bending moment regimes, with increased growth in areas submitted to tension loads in angiosperms^45^. Therefore, thickening growth may be a reaction to these stresses, providing the difference between the elliptical and “inverted-T” shaped sections found in horizontal roots, and the more circular cross-sectional shape in vertical roots. In the LRBs a bending regime may be induced by the self-weight of the tree-bridge system, overturning forces caused by winds in the tree, crossing pedestrians and high forces generated by the flow of water when the bridge is flooded during the monsoon season. It is conceivable that, consistent with the findings reported by Nicoll and Ray^45^ the observed thickening on the lower part of the T-shaped horizontal roots is related to a bending force with a main component in parallel to gravitational force acting on these particular roots, causing tension strains on the lower side of the bent roots and compression strains on their upper side. However, it is likely that bending, torsional and shear forces act in all directions on the horizontal roots within the bridges and differently on different roots or part of roots. This is reflected by the variety of different cross-sectional shapes found among the horizontally trained roots within one bridge. In vertically trained roots, which are presumably mainly submitted to tension or compression forces parallel to the main root axis and to shear forces, development of T-shaped or pronounced elliptical cross-sections by adaptive secondary growth would not present an increase in relevant mechanical stability.

Previous studies of subterranean roots have found I-shaped sections^45,57,58^. These roots resemble I-beams in engineering, optimised for bidirectional flexing in the vertical axis that induce tension and compression on either side of the neutral axis. Studies suggest that I-shaped roots could form under such conditions^59,60^. The lack of I-shaped roots in the living bridges studied so far may be due to the tendency for over-proportional secondary growth on the tension side in angiosperms (well known in the case of reaction wood formation in angiosperm trees)^45,60^. As discussed above, this study is based on a selection of structurally important roots of LRBs. The fact that only elliptic and T-shaped roots were documented does not mean that the existence of I-shaped roots can be ruled out. However, our investigations suggest that I-shaped roots in LRBs – if present – are an exception.

The extraordinary capacity of adaptive secondary growth in aerial roots of *F. elastica* in response to their orientation towards gravitational force and/or the type and extent of mechanical loading, along with their capacity to generate tension stresses by forming tension wood (as reported for *F. benjamina* by Zimmermann *et al*.^36^ and for *F. elastica* by Abasolo *et al*.^37^) can be considered as important selective advantages in the species life cycle as a hemiepiphyte. It is conceivable that in a hemiepiphytic individual of *F. elastica*, each root or part of a root building a scaffold around a host tree’s stem individually adapts its secondary growth to the mechanical strains it is subjected to, thus optimising the final stability of the root network, destined to function as a load-bearing hollow stem after the potential death of the host tree. Their use in the building of bridges of different lengths, from 2 to 53 m reflects their capacity to build mechanically stable networks of different dimensions, according to the size of their host tree and to the position of the germinating seed within the host tree. However, in order to fully understand the functional principles of the adaptive growth processes in aerial roots of *F. elastica* further biological studies have to be carried out, including field work on specimens grown under natural conditions as well as experiments with different designs of mechanical loadings conducted under controlled conditions in greenhouse plants. Such experiments should also generate precise data on aerial root growth related to their age, as in this study age data could only be defined as a rough estimation. Additionally, they should look more precisely at individual root bending regimes as well as at the capacity for tension-side thickening mentioned above.

Furthermore, a variety of aspects of aerial root growth in LRBs still needs clarification. In particular, factors of root history remain unaccounted for. It is still unknown whether builders choose particular roots for horizontal or vertical sections. The point at which the root branches from the tree and the original dimensions of the root may also impact upon the root’s later dimensions. In this regard follow-up studies on the LRBs of Meghalaya could also help to implement concepts of maintenance and construction of new LRBs. Despite the many advantages of living bridges that grow and strengthen themselves, Meghalaya’s Botanical Architecture is menaced with disappearance due to a complex combination of social and environmental factors.

It can be concluded, that beyond their local significance as main components of the unparalleled traditional LRBs in the Khasi and Jaintia Hills of Meghalaya, aerial roots of *Ficus elastica* can also be considered as a unique concept generator for future projects of botanical architecture, which will aim at aligning construction techniques and design aims with the growth phenomena observed in the biological system (compare Ludwig *et al*.^61^, Ludwig^4^, Ludwig *et al*.^39^). However, examining LRBs from a structural engineering perspective also generates a wide range of questions relating to typology, stability, and construction. The structural importance of inosculated joints, the truss-like networks they can form, individual root thickening, and the anchorage of the bridge and parent tree in the ground should be further investigated.

## Supplementary information

Supplementary Information

## Data Availability

The datasets generated and analysed during the current study are available from the corresponding author on reasonable request.
