# Supplementary material for: Living bridges using aerial roots of ficus elastica – an interdisciplinary perspective
Source: Sci Rep. 2019 Aug 22;9:12226. doi: 10.1038/s41598-019-48652-w (PMC6706416; doi:10.1038/s41598-019-48652-w)
Supplement: Supplementary file 1 — Supplementary Information [file 41598_2019_48652_MOESM1_ESM.docx]

**Supplementary Information**

**Living bridges using aerial roots of *Ficus elastica* – an interdisciplinary perspective**

Ferdinand Ludwig^1^*, Wilfrid Middleton^1^, Friederike Gallenmüller^2^, Patrick Rogers^3^, Thomas Speck ^2,4,5^

^1^ Professorship for Green Technologies in Landscape Architecture, Technical University of Munich TUM, 80333 Munich Germany.

^2^ Plant Biomechanics Group, Botanic Garden, University of Freiburg, Freiburg im Breisgau 79104, Germany.

^3^ 34, Minquil Drive, Newark, DE, 19713, U.S.A.

^4^ Freiburg Materials Research Center (FMF), Freiburg im Breisgau 79104, Germany.

^5^ Freiburg Centre for Interactive Materials and Bioinspired Technologies (FIT), Freiburg im Breisgau 79110, Germany.

*Corresponding author: ferdinand.ludwig@tum.de

**Supplementary tables**

Table S1: Bridge data

| **ID** | **Bridge Name** | **Degrees North (WGS84)** | **Degrees East (WGS84)** | **Altitude (m a.m.s.l.)** | **Main Village** | **River** | **Maintenance Group** | **Length (m)** | **Comment** | **Age estimates* (years)** |
| --- | --- | --- | --- | --- | --- | --- | --- | --- | --- | --- |
| 1 | Arch Bridge | 25.337047 | 91.869545 | 727 | Nongblai | - | v | 2 |  |  |
| 2 | Burma 1 | 25.22155 | 91.98137 | 582 | Burma | Wah Rbain | v | 8.8 | span 1  (2 parallel spans) | ca. 70 + |
| 2 | Burma 1 |  |  |  |  |  |  | 9.1 | span 2  (2 parallel spans) | Ca. 40 |
| 3 | Darrang 1 | 25.20516 | 92.01889 | 57 | Darrang | Wah Sheng Pnar | ut | 10.4 |  |  |
| 4 | Darrang Broken | 25.199893 | 92.02186 |  | Darrang | - | ut |  |  |  |
| 5 | Diengsiar 1 | 25.21874 | 91.82688 | 757 | Diengsiar | Wah Juki | uk | 16.9 |  |  |
| 6 | Double-Decker | 25.25135 | 91.67159 | 394 | Nongriat | Mawsaw | v | 24.6 | span 1 (double-decker) | ca. 200 |
| 6 | Double-Decker |  |  |  |  |  |  | 18.4 | span 2 (double decker) |  |
| 7 | Halfway Nongbareh | 25.231476 | 92.02035 | 599 | Nongbareh & Kudeng Rim | Amchrai | uk | 16.2 |  |  |
| 8 | Iar Soh Liang | - | - | - | Rangthylliang | - | i |  |  |  |
| 9 | Kongthong 2 | 25.34485 | 91.82443 | 562 | Kongthong | Wah Langta | uk | 10 |  |  |
| 10 | Kongthong 3 | 25.34468 | 91.82458 | 555 | Kongthong | Wah Langta | uk | 16.3 |  |  |
| 11 | Kudeng Double Decker | 25.22949 | 92.03191 | 542 | Kudeng Rim & Kudeng Thymai | Amlohmar | i | 29 |  |  |
| 12 | Kudeng Rim 5 | 25.22664 | 92.03979 | 520 | Kudeng Rim | Wah Amkshar | v | 30.7 |  |  |
| 13 | Kudeng Rim 8 | 25.23319 | 92.02389 | 673 | Kudeng Rim | Am Sohlashan | ut | 19.2 |  |  |
| 14 | Laitiam 1 | 25.23336 | 91.76718 | 332 | Laitiam | Ustem | v | 19.4 |  |  |
| 15 | Laitiam 2 | 25.2327 | 91.76888 | 307 | Laitiam | Ustem | v | 11.8 |  |  |
| 16 | Long Ti Uyiang | 25.340448 | 91.86933 | 723 | Nongblai | - | v | 12 |  | ca. 70 |
| 17 | Lyngsteng 1 | 25.29853 | 91.80371 | 800 | Lyngsteng | Wah Ulkhit | uk | 14.9 |  |  |
| 18 | Mawkliaw 1 | 25.22774 | 91.81338 | 835 | Mawkliaw | Wah Shari | ut | 12.8 |  |  |
| 19 | Mawkliaw 2 | 25.23001 | 91.81118 | 862 | Mawkliaw | Wah Umlwai | ut | 16.7 |  |  |
| 20 | Mawkyrnot Long Bridge | 25.29576 | 91.88293 | 1070 | Rangthylliang/ Mawkyrnot | Wah Niur | c | 52 |  |  |
| 21 | Mawlam 3 | 25.2627 | 91.84249 | 458 | Rangthylliang/ Mawkyrnot | Wah Mynsaw | i | 13 | span 1 (double-decker) |  |
| 21 | Mawlam 3 |  |  |  |  |  |  | 12.5 | span 2 (double-decker) |  |
| 22 | Mawsaw Hybrid | 25.25766 | 91.67554 |  | Nongriat | Umshiang | v |  |  | ca. 100 |
| 23 | Mawsaw Old | 25.2475 | 91.6745 | 334 | Nongriat | Umshiang | ut | 14.6 |  | ca. 200 |
| 24 | Mawshken 1 | 25.28552 | 91.77377 | 739 | Mawshken | Wah Mdon | uk | 16.9 |  |  |
| **ID** | **Bridge Name** | **Degrees North (WGS84)** | **Degrees East (WGS84)** | **Altitude  (m a.m.s.l.)** | **Main Village** | **River** | **Maintenance Group** | **Length (m)** | **Comment** | **Age estimates* (years)** |
| 25 | Niah Li Bridge | - | - | - | Rangthylliang |  | uk | - |  |  |
| 26 | Nongbah/  Mawshuit 1 | 25.30576 | 91.78537 | 555 | Nongbah/ Mawshuit | Wah Myor | ut | 15 |  |  |
| 27 | Nongbareh 1 (village Link) | 25.22826 | 92.00876 | 607 | Nongbareh | Wah Amlayee/ Ammusai | v | 14.1 |  | ca. 400+ |
| 28 | Nongpriang 3 | 25.27781 | 91.74971 | 495 | Nongpriang | Wah Umshet | ut | 10 |  |  |
| 29 | Nongriat Access | 25.25095 | 91.67219 | 376 | Nongriat | Mawsaw | v | 11.3 |  | ca. 200 |
| 30 | Nongthymmai 1 | 25.2491 | 91.67966 | 407 | Nongthymmai | Simtung | v | 25 |  |  |
| 31 | Nongthymmai 3 | 25.24915 | 91.67992 | 410 | Nongthymmai | Simtung | v | 36.1 |  |  |
| 32 | Nongthymmai Old | 25.25344 | 91.68758 | 528 | Nongthymmai | Simtung | v | 34.1 |  |  |
| 33 | Pdei Kongtim 1 | 25.34547 | 91.81026 | 726 | Pdei/ Kongtim | Umrew (unconfirmed) | v | 33.8 |  |  |
| 34 | Rangthylliang 1 | 25.29647 | 91.88554 | 1211 | Rangthylliang | Wah Kumpa | v | 13.4 |  |  |
| 35 | Rangthylliang 10 | 25.30519 | 91.88525 | 881 | Rangthylliang | Wah Risam | ut | 9.8 |  |  |
| 36 | Rangthylliang 11 | 25.30512 | 91.88543 | 890 | Rangthylliang | Wah Risam | ut | 12.5 |  |  |
| 37 | Rangthylliang 12 | 25.30359 | 91.87199 | 421 | Rangthylliang | Wah Pynursla | uk | 52.7 |  |  |
| 38 | Rangthylliang 13 | 25.30211 | 91.87254 | 447 | Rangthylliang | Pung Stait | uk | 17.5 |  |  |
| 39 | Rangthylliang 2 | 25.29565 | 91.88302 | 1073 | Rangthylliang | Wah Kumpa | c | 9.8 |  |  |
| 40 | Rangthylliang 3 | 25.29692 | 91.88203 | 1053 | Rangthylliang | Yiar Shit Kjat | i | 4.8 |  |  |
| 41 | Rangthylliang 4 | 25.30001 | 91.88274 | 1023 | Rangthylliang | Wah Mawlong | i | 35.7 |  |  |
| 42 | Rangthylliang 5 | 25.30183 | 91.88492 | 1073 | Rangthylliang | Wah Sohshiat | ut | 13.3 |  |  |
| 43 | Rangthylliang 6 | 25.30676 | 91.88983 | 1149 | Rangthylliang | Wah Pynursla | i | 12.3 |  | ca. 70 |
| 44 | Rangthylliang 7 | 25.30684 | 91.88773 | 1029 | Rangthylliang | Wah Pynursla | v | 18.8 |  |  |
| 45 | Rangthylliang 8 | 25.30638 | 91.88557 | 891 | Rangthylliang | Wah Pynursla | i | 18.3 |  |  |
| 46 | Rangthylliang/ Mawkyrnot 2 | 25.29566 | 91.8829 | 1068 | Rangthylliang | Wah Niur | c | 40.7 |  |  |
| 47 | Rimai Bridge | 25.19 | 91.92 | 280 | Rimai | Wah Kwang | v | 31.8 |  |  |
| 48 | Rymmai 1 | 25.29749 | 91.77993 | 483 | Rymmai | Wah Umlwai | uk | 18.5 |  |  |
| 49 | Rynsiet | 25.2779 | 91.62988 | 354 | Nongsteng and Nongbah | Rynsiet | v | 35.6 |  |  |
| 50 | Siej | 25.213028 | 91.6768 | 664 | Siej | Umkar | i | 25.2 |  | 66 |
| 51 | Sohkhmi 1 | 25.25058 | 91.78358 | 418 | Sohkhmi | - | uk | 11.2 |  |  |
| 52 | Suktia 1 | 25.22289 | 91.79153 | 177 | Suktia | Wah Pohwer | ut | 9.2 |  |  |
| 53 | Thangkyrta 1 | 25.30708 | 91.80974 | 706 | Thangkyrta | - | ut | 6.4 |  |  |
| 54 | Thangkyrta 2 | 25.30624 | 91.80693 | 774 | Thangkyrta | Wah Umsong (unconfirmed) | uk | 20.4 |  |  |
| 55 | Tynrong 1 | 25.24808 | 91.63512 | 443 | Tynrong | - | uk | 14 |  |  |
| 56 | Tyrngei 1 | 25.2383 | 91.79356 | 331 | Tyrngei | Wah Umsha | uk | 11.4 |  |  |
| 57 | Ummonoi | 25.20834 | 91.67049 | 689 | Soh Sarat | Ummonoi | c | 7 |  |  |
| **ID** | **Bridge Name** | **Degrees North (WGS84)** | **Degrees East (WGS84)** | **Altitude  (m a.m.s.l.)** | **Main Village** | **River** | **Maintenance Group** | **Length (m)** | **Comment** | **Age estimates* (years)** |
| 58 | Wah Amlohmar | 25.23757 | 92.03035 | 706 | Kudeng Rim | Amlohmar | ut | 20.2 |  |  |
| 59 | Wah Kdal | 25.33484 | 91.86614 | 678 | Nongblai | Wah Kdal | i | 8 |  | ca. 35 |
| 60 | Wah Koh La 1 | 25.24098 | 91.66119 | 278 | Myntheng | Koh La | c | 19.3 |  | ca. 60 |
| 61 | Wah Koh La 2 | 25.24095 | 91.66093 | 277 | Myntheng | Koh La | c | 15.4 |  | ca. 200 |
| 62 | Wah Lar Ung | 25.345118 | 91.87331 | 762 | Nongblai | Lar Ung | i | 18.3 |  | ca. 700 |
| 63 | Wah Lyngkhen Hybrid | 25.340519 | 91.87037 | 732 | Nongblai | Lyngkhen | v | 10 |  |  |
| 64 | Wah Lynseng | 25.34301 | 91.86846 | 780 | Nongblai | Lynseng | v | 34.1 |  |  |
| 65 | Wah Matieh Lower | 25.339943 | 91.87265 | 788 | Nongblai | Matieh | v | 6.3 |  |  |
| 66 | Wah Matieh Upper | 25.339943 | 91.87268 | 790 | Nongblai | Matieh | v | 10 |  |  |
| 67 | Wah Shoh Klea | 25.19 | 91.89 | 350 | Lyngkhong | Shoh Klea | uk | 16.3 |  |  |
| 68 | Wah Soh Mad | - | - | - | Rangthylliang | Wah Soh Mad | i | - |  |  |
| 69 | Wah Soh Shiat | - | - | - | Rangthylliang | Wah Soh Shiat | uk | - |  |  |
| 70 | Wah Spit | 25.33619 | 91.86714 | 718 | Nongblai | Spit | v | 14.6 |  |  |
| 71 | Wah Surah | 25.344595 | 91.874176 | 756 | Nongblai | Surah | v | 6.5 |  |  |
| 72 | Wah Thyllong | 25.20688 | 91.89737 | 541 | Mawlynnong/ Nowhet/ Riwai | Thyllong | c | 13 |  | ca. 200+ |
| 73 | Wah Tiah Long | 25.33727 | 91.87263 | 830 | Nongblai | Tieh Long | i | 6.2 |  | ca. 15 |
| 74 | Wah Tumbai | 25.339113 | 91.869484 | 721 | Nongblai | Tumbai | v | 10.08 |  |  |
| 75 | Wah Um Thliem | 25.340513 | 91.8721 | 737 | Nongblai | Um Thliem | v | 8 |  |  |
| 76 | Nongthymmai 2 | 25.24914 | 91.67968 | 407 | Nongthymmai | Simtung | v | 4 | new attempt, tourism interest |  |

Abbreviations for maintenance groups:
i - individual or family
v - village or community
c - consortium of villages/community groups
uk – unknown
ut - untended

Where geolocation disagrees between measurements by PR and WM, online maps were used to pick best match from two locations.
Much of the data and many images of bridges documented by P.R. are available online on the website by P.R. https://livingrootbridges.com

*Ages of the bridges refer to the years when this study was conducted (2017/2018).

Table S2: Root morphology data

| **Orientation** | **h (cm)  (major axis)** | **d (cm)  (minor axis, resp. mean minor axis)*** | **d1 (cm) (minor axis,**  **smallest width)** | **d2 (cm) (minor axis,**  **largest width)** | **CSA   (cm^2^)** | **Ratio h/d** | **Ratio d_2_/d_1_ (T-Ratio)** | **Root Age (years)** |
| --- | --- | --- | --- | --- | --- | --- | --- | --- |
| Horizontal | 30,0 | 14,0 | 13,0 | 15,0 | 329,87 | 2,14 | 1,15 |  |
| Horizontal | 40,0 | 13,5 | 12,0 | 15,0 | 424,12 | 2,96 | 1,25 |  |
| Horizontal | 21,0 | 9,0 | 8,0 | 10,0 | 148,44 | 2,33 | 1,25 |  |
| Horizontal | 20,0 | 7,0 | 6,0 | 8,0 | 109,96 | 2,86 | 1,33 |  |
| Horizontal | 75,0 | 16,0 | 13,0 | 19,0 | 942,48 | 4,69 | 1,46 |  |
| Horizontal | 33,0 | 10,0 | 8,0 | 12,0 | 259,18 | 3,30 | 1,50 |  |
| Horizontal | 20,0 | 15,0 | 12,0 | 18,0 | 235,62 | 1,33 | 1,50 |  |
| Horizontal | 30,0 | 12,5 | 10,0 | 15,0 | 294,52 | 2,40 | 1,50 |  |
| Horizontal | 30,0 | 15,5 | 12,0 | 19,0 | 365,21 | 1,94 | 1,58 |  |
| Horizontal | 40,0 | 20,0 | 15,0 | 25,0 | 628,32 | 2,00 | 1,67 |  |
| Horizontal | 60,0 | 22,5 | 15,0 | 30,0 | 1060,29 | 2,67 | 2,00 |  |
| Horizontal | 47,5 | 10,0 | 5,0 | 15,0 | 373,06 | 4,75 | 3,00 |  |
| Horizontal | 32,5 | 11,0 | 5,0 | 17,0 | 280,78 | 2,95 | 3,40 |  |
| Vertical | 1,6 | 1,6 |  |  | 2,01 | 1,00 |  |  |
| Vertical | 1,6 | 1,6 |  |  | 2,01 | 1,00 |  |  |
| Horizontal | 1,6 | 1,6 |  |  | 2,01 | 1,00 |  |  |
| Horizontal | 1,9 | 1,9 |  |  | 2,84 | 1,00 |  |  |
| Horizontal | 2,0 | 2,0 |  |  | 3,14 | 1,00 |  | 7 |
| Horizontal | 2,1 | 2,1 |  |  | 3,46 | 1,00 |  |  |
| Vertical | 2,1 | 2,1 |  |  | 3,46 | 1,00 |  | 7 |
| Horizontal | 2,2 | 2,2 |  |  | 3,80 | 1,00 |  |  |
| Vertical | 2,2 | 2,2 |  |  | 3,80 | 1,00 |  | 18 |
| Horizontal | 2,4 | 2,4 |  |  | 4,52 | 1,00 |  |  |
| Horizontal | 2,5 | 2,5 |  |  | 4,91 | 1,00 |  |  |
| Vertical | 2,5 | 2,5 |  |  | 4,91 | 1,00 |  |  |
| Horizontal | 2,5 | 2,5 |  |  | 4,91 | 1,00 |  | 18 |
| Horizontal | 2,5 | 2,5 |  |  | 4,91 | 1,00 |  | 18 |
| Vertical | 2,5 | 2,5 |  |  | 4,91 | 1,00 |  | 18 |
| Horizontal | 2,7 | 2,7 |  |  | 5,73 | 1,00 |  |  |
| Horizontal | 2,7 | 2,7 |  |  | 5,73 | 1,00 |  |  |
| Vertical | 2,8 | 2,8 |  |  | 6,16 | 1,00 |  | 66 |
| Horizontal | 2,9 | 2,9 |  |  | 6,61 | 1,00 |  |  |
| Horizontal | 2,9 | 2,9 |  |  | 6,61 | 1,00 |  |  |
| Horizontal | 2,9 | 2,9 |  |  | 6,61 | 1,00 |  |  |
| Horizontal | 2,9 | 2,9 |  |  | 6,61 | 1,00 |  |  |
| Horizontal | 2,9 | 2,9 |  |  | 6,61 | 1,00 |  |  |
| Vertical | 3,0 | 3,0 |  |  | 7,07 | 1,00 |  |  |
| Horizontal | 3,0 | 3,0 |  |  | 7,07 | 1,00 |  |  |
| Horizontal | 3,0 | 3,0 |  |  | 7,07 | 1,00 |  |  |
| Horizontal | 3,0 | 3,0 |  |  | 7,07 | 1,00 |  |  |
| Horizontal | 3,0 | 3,0 |  |  | 7,07 | 1,00 |  | 7 |
| **Orientation** | **h (cm)  (major axis)** | **d (cm)  (minor axis, resp. mean minor axis)*** | **d1 (cm) (minor axis,**  **smallest width)** | **d2 (cm) (minor axis,**  **largest width)** | **CSA  (cm^2^)** | **Ratio h/d** | **Ratio d_2_/d_1_ (T-Ratio)** | **Root Age (years)** |
| Vertical | 3,0 | 3,0 |  |  | 7,07 | 1,00 |  | 7 |
| Vertical | 3,0 | 3,0 |  |  | 7,07 | 1,00 |  | 7 |
| Vertical | 3,0 | 3,0 |  |  | 7,07 | 1,00 |  | 66 |
| Vertical | 3,2 | 3,2 |  |  | 8,04 | 1,00 |  |  |
| Vertical | 3,2 | 3,2 |  |  | 8,04 | 1,00 |  |  |
| Vertical | 3,2 | 3,2 |  |  | 8,04 | 1,00 |  | 7 |
| Vertical | 3,2 | 3,2 |  |  | 8,04 | 1,00 |  | 66 |
| Vertical | 3,3 | 3,3 |  |  | 8,55 | 1,00 |  | 7 |
| Horizontal | 3,5 | 3,5 |  |  | 9,62 | 1,00 |  |  |
| Horizontal | 3,5 | 3,5 |  |  | 9,62 | 1,00 |  | 18 |
| Horizontal | 3,5 | 3,5 |  |  | 9,62 | 1,00 |  | 18 |
| Horizontal | 3,5 | 3,5 |  |  | 9,62 | 1,00 |  | 66 |
| Vertical | 3,5 | 3,5 |  |  | 9,62 | 1,00 |  | 66 |
| Horizontal | 3,7 | 3,7 |  |  | 10,75 | 1,00 |  |  |
| Horizontal | 4,0 | 4,0 |  |  | 12,57 | 1,00 |  |  |
| Horizontal | 4,0 | 4,0 |  |  | 12,57 | 1,00 |  | 7 |
| Horizontal | 4,0 | 4,0 |  |  | 12,57 | 1,00 |  | 7 |
| Vertical | 4,0 | 4,0 |  |  | 12,57 | 1,00 |  | 18 |
| Vertical | 4,0 | 4,0 |  |  | 12,57 | 1,00 |  | 18 |
| Vertical | 4,0 | 4,0 |  |  | 12,57 | 1,00 |  | 18 |
| Vertical | 4,1 | 4,1 |  |  | 13,20 | 1,00 |  |  |
| Vertical | 4,1 | 4,1 |  |  | 13,20 | 1,00 |  |  |
| Vertical | 4,5 | 4,5 |  |  | 15,90 | 1,00 |  |  |
| Horizontal | 4,5 | 4,5 |  |  | 15,90 | 1,00 |  | 66 |
| Vertical | 4,6 | 4,6 |  |  | 16,62 | 1,00 |  |  |
| Vertical | 4,9 | 4,9 |  |  | 18,86 | 1,00 |  |  |
| Horizontal | 4,9 | 4,9 |  |  | 18,86 | 1,00 |  |  |
| Horizontal | 5,0 | 5,0 |  |  | 19,63 | 1,00 |  |  |
| Horizontal | 5,0 | 5,0 |  |  | 19,63 | 1,00 |  | 66 |
| Horizontal | 5,0 | 5,0 |  |  | 19,63 | 1,00 |  | 66 |
| Horizontal | 5,2 | 5,2 |  |  | 21,24 | 1,00 |  | 66 |
| Vertical | 5,6 | 5,6 |  |  | 24,63 | 1,00 |  |  |
| Vertical | 5,7 | 5,7 |  |  | 25,52 | 1,00 |  |  |
| Vertical | 5,9 | 5,9 |  |  | 27,34 | 1,00 |  |  |
| Vertical | 6,0 | 6,0 |  |  | 28,27 | 1,00 |  |  |
| Horizontal | 6,4 | 6,4 |  |  | 32,17 | 1,00 |  |  |
| Vertical | 6,5 | 6,5 |  |  | 33,18 | 1,00 |  |  |
| Vertical | 8,0 | 8,0 |  |  | 50,27 | 1,00 |  |  |
| Vertical | 8,8 | 8,8 |  |  | 60,82 | 1,00 |  |  |
| Vertical | 8,9 | 8,9 |  |  | 62,21 | 1,00 |  |  |
| Vertical | 10,0 | 10,0 |  |  | 78,54 | 1,00 |  |  |
| Horizontal | 10,0 | 10,0 |  |  | 78,54 | 1,00 |  |  |
| Vertical | 10,5 | 10,5 |  |  | 86,59 | 1,00 |  |  |
| Vertical | 11,6 | 11,6 |  |  | 105,68 | 1,00 |  |  |
| **Orientation** | **h (cm)  (major axis)** | **d (cm)  (minor axis, resp. mean minor axis)*** | **d1 (cm) (minor axis,**  **smallest width)** | **d2 (cm) (minor axis,**  **largest width)** | **CSA  (cm^2^)** | **Ratio h/d** | **Ratio d_2_/d_1_ (T-Ratio)** | **Root Age (years)** |
| Vertical | 11,6 | 11,6 |  |  | 105,68 | 1,00 |  |  |
| Horizontal | 15,0 | 15,0 |  |  | 176,71 | 1,00 |  |  |
| Horizontal | 2,0 | 1,5 |  |  | 2,36 | 1,33 |  | 7 |
| Vertical | 2,4 | 2,1 |  |  | 3,96 | 1,14 |  |  |
| Horizontal | 3,0 | 2,5 |  |  | 5,89 | 1,20 |  | 7 |
| Horizontal | 3,0 | 2,5 |  |  | 5,89 | 1,20 |  | 7 |
| Horizontal | 3,5 | 3,0 |  |  | 8,25 | 1,17 |  | 7 |
| Horizontal | 4,0 | 3,8 |  |  | 11,94 | 1,05 |  | 18 |
| Vertical | 4,1 | 3,4 |  |  | 10,95 | 1,21 |  |  |
| Vertical | 4,5 | 3,0 |  |  | 10,60 | 1,50 |  | 18 |
| Vertical | 4,7 | 3,8 |  |  | 14,03 | 1,24 |  |  |
| Horizontal | 5,3 | 4,8 |  |  | 19,98 | 1,10 |  | 7 |
| Horizontal | 5,3 | 3,5 |  |  | 14,57 | 1,51 |  | 7 |
| Vertical | 5,5 | 5,0 |  |  | 21,60 | 1,10 |  | 18 |
| Vertical | 5,8 | 5,4 |  |  | 24,60 | 1,07 |  |  |
| Vertical | 6,0 | 5,5 |  |  | 25,92 | 1,09 |  |  |
| Horizontal | 6,0 | 4,5 |  |  | 21,21 | 1,33 |  | 66 |
| Vertical | 6,1 | 4,5 |  |  | 21,56 | 1,36 |  |  |
| Horizontal | 6,5 | 5,5 |  |  | 28,08 | 1,18 |  | 66 |
| Horizontal | 6,5 | 4,0 |  |  | 20,42 | 1,63 |  | 7 |
| Horizontal | 7,0 | 5,0 |  |  | 27,49 | 1,40 |  | 18 |
| Vertical | 7,2 | 4,9 |  |  | 27,71 | 1,47 |  |  |
| Horizontal | 7,5 | 5,0 |  |  | 29,45 | 1,50 |  | 7 |
| Horizontal | 7,5 | 4,5 |  |  | 26,51 | 1,67 |  | 18 |
| Horizontal | 7,5 | 4,5 |  |  | 26,51 | 1,67 |  | 7 |
| Vertical | 7,7 | 5,1 |  |  | 30,84 | 1,51 |  |  |
| Horizontal | 8,5 | 5,0 |  |  | 33,38 | 1,70 |  | 18 |
| Horizontal | 9,0 | 5,5 |  |  | 38,88 | 1,64 |  | 66 |
| Horizontal | 9,5 | 5,0 |  |  | 37,31 | 1,90 |  | 66 |
| Horizontal | 10,0 | 7,0 |  |  | 54,98 | 1,43 |  | 18 |
| Horizontal | 10,0 | 4,5 |  |  | 35,34 | 2,22 |  | 18 |
| Vertical | 10,2 | 9,7 |  |  | 77,71 | 1,05 |  |  |
| Vertical | 10,3 | 8,7 |  |  | 70,38 | 1,18 |  |  |
| Horizontal | 11,0 | 7,0 |  |  | 60,48 | 1,57 |  | 18 |
| Horizontal | 11,0 | 5,0 |  |  | 43,20 | 2,20 |  | 18 |
| Horizontal | 12,5 | 7,5 |  |  | 73,63 | 1,67 |  | 18 |
| Vertical | 13,0 | 9,1 |  |  | 92,91 | 1,43 |  |  |
| Horizontal | 13,0 | 7,0 |  |  | 71,47 | 1,86 |  | 66 |
| Horizontal | 13,4 | 13,1 |  |  | 137,87 | 1,02 |  | 15 |
| Horizontal | 13,5 | 6,5 |  |  | 68,92 | 2,08 |  | 66 |
| Vertical | 14,5 | 9,1 |  |  | 103,63 | 1,59 |  |  |
| Horizontal | 15,0 | 12,0 |  |  | 141,37 | 1,25 |  | 66 |
| Vertical | 15,0 | 8,5 |  |  | 100,14 | 1,76 |  |  |
| Vertical | 15,5 | 9,4 |  |  | 114,43 | 1,65 |  |  |
| **Orientation** | **h (cm)  (major axis)** | **d (cm)  (minor axis, resp. mean minor axis)*** | **d1 (cm) (minor axis,**  **smallest width)** | **d2 (cm) (minor axis,**  **largest width)** | **CSA  (cm^2^)** | **Ratio h/d** | **Ratio d_2_/d_1_ (T-Ratio)** | **Root Age (years)** |
| Horizontal | 16,0 | 7,0 |  |  | 87,96 | 2,29 |  | 18 |
| Horizontal | 16,5 | 8,5 |  |  | 110,15 | 1,94 |  | 18 |
| Horizontal | 20,0 | 10,0 |  |  | 157,08 | 2,00 |  |  |
| Horizontal | 27,0 | 8,0 |  |  | 169,65 | 3,38 |  |  |
| Horizontal | 30,0 | 10,0 |  |  | 235,62 | 3,00 |  |  |
| Horizontal | 33,0 | 15,0 |  |  | 388,77 | 2,20 |  |  |
| Horizontal | 38,0 | 12,0 |  |  | 358,14 | 3,17 |  |  |
| Horizontal | 40,0 | 12,0 |  |  | 376,99 | 3,33 |  |  |
| Horizontal | 40,0 | 12,0 |  |  | 376,99 | 3,33 |  |  |
| Horizontal | 60,0 | 20,0 |  |  | 942,48 | 3,00 |  |  |
| Horizontal | 80,0 | 15,0 |  |  | 942,48 | 5,33 |  |  |

*For elliptical roots d was measured, for T-shaped roots d was calculated as the mean of d1 and d2.
